# Pyroptosis and respiratory diseases: A review of current knowledge

**DOI:** 10.3389/fimmu.2022.920464

**Published:** 2022-09-30

**Authors:** Jialiang Sun, Yanan Li

**Affiliations:** Department of Pediatrics, The First Hospital of Jilin University, Changchun, China

**Keywords:** pyroptosis, caspase, NLRP3, respiratory diseases, inflammation, infection

## Abstract

Pyroptosis is a relatively newly discovered programmed cell death accompanied by an inflammatory response. In the classical view, pyroptosis is mediated by caspases-1,-4,-5,-11 and executed by GSDMD, however, recently it was demonstrated that caspase-3 and-8 also participate in the process of pyroptosis, by cleaving GSDMD/E and GSDMD respectively. Different from autophagy and apoptosis, many pores are formed on the cell membrane during pyroptosis, which makes the cell membrane lose its integrity, eventually leading to the release of cytokines interleukin(IL)-1β and IL-18. When the body is infected with pathogens or exposed to some stimulations, pyroptosis could play an immune defense role. It is found that pyroptosis exists widely in infectious and inflammatory respiratory diseases such as acute lung injury, bronchial dysplasia, chronic obstructive pulmonary disease, and asthma. Excessive pyroptosis may accompany airway inflammation, tissue injury, and airway damage, and induce an inflammatory reaction, leading to more serious damage and poor prognosis of respiratory diseases. This review summarizes the relationship between pyroptosis and related respiratory diseases.

## Introduction

When the host is attacked by exogenous and/or endogenous pathogenic microorganisms, cells can clear infected cells by programmed cell death. The specific ways of host cell programmed death induced by pathogens include apoptosis, autophagy necrosis, etc. (1) When there is a microbial infection or non-infectious stimulation, the intracellular pattern recognition receptors (PRR) initiate a series of signal cascade reactions to execute the effector protein Gasdermin D (GSDMD) by recognizing pathogen-related molecular patterns (PAMPs) and damage-related molecular patterns (DAMPs) and induce cell pyroptosis, which is also considered to be one of the innate immune defense mechanisms of the host against intracellular pathogen infection (2). The PAMP detection uses a distinct set of receptors to detect the presence of molecular patterns that originate from infections. However, pathogens are often able to bypass the triggering of the innate immune system through the loss of PAMPs, structural modifications of PAMPs, etc. DAMPs are an alternative system that is still based on a simple molecule recognition model, but the molecular patterns originate from cellular death caused by pathogens. Distinct from directly detecting molecular patterns, it was recently found that the inflammasome components NLRP3 (NOD-, leucine-rich repeats (LRR)- and pyrin domain-containing 3) and pyrin act as signal integrators that are capable of detecting perturbations (eg., triggered by infection) in cytoplasmic homeostasis. Monitoring these perturbations (e.g., the phosphorylation of pyrin inflammasome), which was termed “homeostasis-altering molecular processes” (HAMPs) by Liston et al. (3), HAMPs sensing provides the innate immune system potent flexibility in detecting evolutionarily novel infections. However, HAMPs may also elevate the risk of inflammatory diseases (3). Zychlinsky et al. (4) first discovered the phenomenon of pyroptosis in 1992. They found the morphological changes in macrophages infected with *Shigella flexneri*: DNA fragments, vacuole formation, chromatin pyknosis and dependence on caspase-1 activity. Then in 1996, Monack et al. (5) found a similar phenomenon in mammalian epithelial cells and macrophages infected with invasive *Salmonella typhimurium*. Soon after, in 1997, Hilbi et al. (6) found that *Shigella flexneri* induces PCD in macrophages with mature interleukin(IL)-1β releasing specifically. In 1999, Hersh et al. (7) found that Caspase-1 was essential for the macrophage apoptosis induced by *Salmonella* spp. However, this mode of cell death characterized by Caspase-1 dependence and morphological changes (containing DNA fragments, vacuole formation, chromatin pyknosis) was considered to be apoptosis at that time. In 2001, Cookson and Brennan (8) first used the concept of pyroptosis to define the unique caspase-1-dependent pattern of cell death in macrophages for the treatment of *salmonella* infection. Similar to apoptosis, pyroptosis also showed nuclear condensation, chromatin DNA fragmentation, positive TUNEL staining, and positive AnnexinV staining. However, different from apoptosis, GSDMD forms large, ~21 nanometer (nm)-diameter pores in the plasma membrane to drive pyroptosis. These pores are sufficiently large to allow the leakage of intercellular contents smaller than their size and the inflow of extracellular water, making the cell membrane lose its integrity and the ability to regulate the entry/exit of substances, eventually leading to the dissolution of the cell membrane, the release of cell contents and the induction of inflammation (9). At the same time, cells release IL-1β and IL-18 during pyroptosis, which can recruit more inflammatory cells and expand the inflammatory response (10). Moderate pyroptosis can help to combat the invasion of pathogens and inflammatory factors, and helps to maintain the stability of the internal environment. However, excessive pyroptosis overactivates the immune response and plays an important role in both infection and inflammation, causing cell and tissue damage, and leading to diseases.

Acute and chronic respiratory diseases are threats to human health, the prevalence of respiratory diseases is increasing in the context of deteriorating air quality (11, 12). Pyroptosis exists widely in the occurrence of respiratory diseases as a relatively newly discovered pattern of inflammatory programmed cell death, such as acute lung injury (ALI), bronchial dysplasia, chronic obstructive pulmonary disease (COPD), asthma, etc. However, the above diseases have not been completely conquered. In the case of asthma, the symptoms of most patients can be controlled based on corticosteroids, but there are still some patients whose treatment is ineffective, and the side effects of long-term use mean that alternative treatments should be sought. Theoretically, inhibiting the occurrence of many kinds of respiratory system-related cell death can reduce the degree of lung cell injury, reduce the production of inflammatory factors and reduce the inflammatory reaction. Some studies have shown promising and beneficial results, providing new possible therapeutic targets for the diseases (13–16). Therefore, it is necessary to further study the effects of pyroptosis in the field of respiratory diseases. This review intends to summarize the relationship between pyroptosis and respiratory diseases, to explore potential and effective clinical treatments.

## 1 The mechanism of pyroptosis

### 1.1 Molecular mechanism of pyroptosis

There are several pathways involved in pyroptosis, including classical pyroptosis signaling pathway induced by Caspase-1 activation, Caspase-4/5/11-mediated non- classical pyroptosis signaling pathway, Caspase-3-dependent pyroptosis signaling pathway and Caspase-8-dependent pyroptosis signaling pathway, etc. Although the pyroptosis process is initiated by different pathways, it is finally accomplished by the GSDM family which has been shown to have the ability to form pores in the cell membrane. Most members of the GSDM family share the highly conserved N-terminal domain and C-terminal domain, including GSDM A, B, C, D, E, and PJVK/DFNB59 (17). N-terminal domain has lipophilic properties, specifically binds to phosphatidylinositol inside the cell membrane and cardiolipin outside the bacterial plasma membrane (18, 19). The N-terminal domain is activated by the dissociation of the C-terminal domain to form pores on the membrane which cause osmotic imbalance and the leakage of intercellular contents, inducing inflammation (19). The relatively well-studied GSDM family members are GSDMD and GSMDE, apart from them, GSDMA is widely expressed in the skin, it was reported that the GSDMA is specifically cleaved by the SpeB protease of *group A Streptococcus*, which triggered keratinocyte pyroptosis (20). The cleaved and full-length GSDMB could bind to membrane lipids, distinguishing them from other GSDMs, however, it is GSDMB N-terminal but not the full-length GSDMB that induces pyroptosis (21, 22). Besides, the cleavage of GSDMC was found driven by caspase-8 to induce pyroptosis. In 2021, Zhang et al. demonstrated that the metabolite α-ketoglutarate induced GSDMC-dependent pyroptosis through caspase-8 activated by death receptor-6 (23). However, the pyroptotic mechanism and signaling pathways of the GSDM family remain unclear, which deserves further studied in the future.

GSDMD protein, a member of the GSDM family which is the most researched, is a common substrate of inflammatory caspase (24). GSDMD is not only a common substrate of inflammatory caspase but also an effector molecule of pyroptosis (19, 25). When the host cell is stimulated by endogenous or exogenous factors, the intracellular PRR recognizes and binds the corresponding ligands to form a cytoplasmic polyprotein complex, which activates inflammatory caspase-1 and caspase-4/5/11 to cleave GSDMD, and binds to the lipids on the cell membrane to form pores, subsequently, inflammatory cytokines IL-1β and IL-18 are released from cells. IL-1β is a thermogenic cytokine that signals through IL-1 receptor 1 (IL-1R1). It can induce fever, stimulate leukocytes to express various cytokines (such as IL-6) and chemokine (such as IL-18), promote leukocyte migration to organs, and activate immune cells, thus exaggerating the development of inflammation (26). The biological function of IL-18 is different from that of IL-1β. Extracellular IL-18 binds and activates IL-18 receptors to promote T helper cell type 1 (Th1) or Th2 immune response: IL-18 induces Th1 cell response and IFN- γ production, which plays a key role in intracellular elimination of pathogens. IL-18 also plays a role in the differentiation of Th2 cells, and experimental models and clinical data indicated that it is involved in the occurrence of allergic airway inflammation (27, 28).

#### 1.1.1 Classical pyroptosis pathway

Caspase-1 is the first member of the Caspase family, the pyroptosis pathway is triggered by the activation of Caspase-1 and a series of recognition receptors of inflammasomes. The inflammasome consists of a sensor protein, an adaptor, and pro-caspase-1 (29). PRRs act as sensor proteins that detect signals to induce inflammatory responses, nod-like receptors (NLRs) are the best known and most studied receptors among PRRs (30). NLRs can identify PAMPs such as fungi, bacteria, and viruses, and risk factors released by endogenous cellular injuries such as cholesterol, mitochondrial damage, and oxidative stress (31, 32). According to the different components of NLR proteins, NLRs can be divided into NLR family pyrin domain protein 1 (NLRP1), NLRP3, NLRC4, AIM2, IPAF inflammasome, etc. These NLRs are characterized by the presence of a central nucleotide-binding and oligomerization (NACHT) domain that is commonly flanked by carboxy-terminal LRRs and amino-terminal caspase recruitment domain (CARD) or pyrin domains (PYD) (10). The PYD interacts with apoptosis-associated speck-like protein containing a CARD (ASC) which in turn binds pro-caspase-1. ASC is an adaptor protein used by many NLRs to connect pro-caspase-1 and the inflammasomes through its CARD domain. Both NLRP1 and NLRC4 contain a CARD domain distinguishing them from other inflammasomes, can directly recruit pro-caspase-1 with no need for ASC (33).

NLRP3 inflammasome responds to bacteria, viruses, and fungi, and is also found to be closely related to aseptic inflammation (34). The activation of the inflammasome is a key step in the production of inflammatory factors IL-1 β and 1L-18. Studies have shown that the activation of NLRP3 requires two signal processes: the first is the initiation process. After the NLRP3 inflammasome recognizes PAMPs or DAMPs, the activation of nuclear factor-kB (NF-kB) stimulates the initiation signal to promote the expression of NLRP3, pro-inter-leukin-1β, pro-IL-1β) and pro-IL-18. The second is the inflammasome assembly and activation process: damage-related molecular mode activates NLRP3, unlike NLRP1, NLRP3 does not contain a CARD domain, but instead recruits pro-caspase-1 through ASC that connects NLRP3 and caspase-1. As for other inflammasomes, humans have only one NLRP1 protein, but the mouse NLRP1 protein is polymorphic encoding 3 paralogs: NLRP1a, 1b, and 1c (35). The mouse NLRP1b and rat NLRP1 inflammasome sensors are activated following their cleavage by gram-positive bacterium *Bacillus anthracis* containing lethal factors (36). Besides, human NLRP1 was found as a direct sensor for dsRNA and thus RNA virus infection (37). NLRC4 inflammasome is activated by NAIP family proteins, responds to cytosolic flagellin, and the inner rod and needle proteins of the type III secretion system of bacteria (38). AIM2 is activated following direct binding to cytoplasmic dsDNA (39). Pyrin is activated by assembling microtubules and responds to bacterial toxin-induced modifications of Rho GTPases (40). Ultimately, activation of the inflammasomes leads to the activation of caspase-1, and then activates pro-IL-1 β and pro-IL-18 into IL-1 β and IL-18. Caspase-1 cleaves GSDMD and divides GSDMD protein into lipophilic N-terminal domain and hydrophilic C-terminal domain, in which the N-terminal domain is released from the C-terminal inhibitory domain, which promotes the release of downstream inflammatory cytokines IL-1β and IL-18, recruits more inflammatory cells, and expands the inflammatory response (41, 42). The formation of the inflammasome is a key link in pyroptosis, which can affect the assembly and activation of inflammatory factors and play an important role in the regulation of pyroptosis.

#### 1.1.2 Non-classical pyroptosis pathway

The caspase-1-independent pyroptosis pathway is called the non-classical pyroptosis pathway. Studies have shown that there are still other non-classical pyroptosis pathways in biology. Human Caspase-4, Caspase-5, and mouse Caspase-11 was believed to be directly linked to the LPS of Gram-negative bacteria (such as Escherichia coli, Salmonella typhimurium, Shigella flexneri, and Burkholderia thailandensis). When mice are infected by Gram-negative bacteria, LPS directly binds to the CARD of Caspase-11, thus activating Caspase-11, the activating progress of human Caspase-4 and Caspase-5 is the same as that of caspase-11 (43). Although the function of human caspase-5 has been shown similar to human caspase-4, there is few studies in respect to LPS recognition by caspase-5. Inflammatory caspases-4/11 can directly bind the lipid A moiety of LPS, however, how LPS that sequestered in the membranes of cytosol-invading bacteria activates non-classical caspases remains not fully understood. In classical pyroptosis pathway, inflammasomes require an adaptor to link sensors to caspase-1 activation in classical pyroptosis pathway, hence researchers anticipated that some CARD-containing proteins may similarly serve as an LPS upstream sensor and a caspase-11 activator (44). However, it was observed that caspase-11 was not activated after co-expression of 18 different CARD-containing proteins, which suggested that such a protein may not be necessary (45). Recently, guanylate-binding proteins (GBPs) were proposed that they may be essential for LPS/lipid A sensing (46).`IFN inducible GTPases, including GBPs and immunity related GTPases modulate both cell-autonomous and innate immunity against pathogens (Gram-negative bacteria,virus, and so on), and promote human Caspase-4 and mouse Caspase-11 responses upon transfection of LPS, which play a crucial role in immune defense against pathogens (47). Activation of GBPs also contributes to IL-1β and IL-18 processing and secretion during infection, and induces pyroptosis through activating the NLRP3 inflammasome and promoting LPS release into the host cells cytosol (48). Recently, several GBP family members have been implicated to serve as LPS receptors that recruit the non-classical inflammasome caspases to cytosolic bacteria (49, 50). GBPs were found to control Caspase-4 activation hierarchically: After targeting the Gram-negative bacterial surface, GBP1 recruits GBP2, GBP3 and GBP4 to create a stabilized GBPs oligomeric structure, GBP2 and GBP4 control caspase-4 recruitment to the LPS-rich bacterial surface, and GBP3 governs caspase-4 activation (51). Besides, it was indicated that GBPs contributed to the LPS uptake from membrane interfaces and be involved in the activation of caspase-11 and GSDMD *in vivo* (48, 49). Therefore, activation of caspase-4/11 through the GBPs platform is essential to induce GSDMD-dependent pyroptosis in response to cytosol-invading bacteria, thereby contributing to infection restriction. In addition, Kayagaki (52) and his colleagues found that Caspase-11-mediated non-classical pyroptosis can activate caspase-1 alone in the absence of NLRP3 and ASC, leading to the maturation of IL-1β precursors. In addition, the non-classical pyroptosis pathway of human monocytes involved in GSDMB can directly bind to the CARD of caspase-4, enhancing non-classical pyroptosis by promoting the activation of caspase-4 by LPS (22).

#### 1.1.3 Caspase-3-dependent pyroptosis pathway

**Figure 1:**
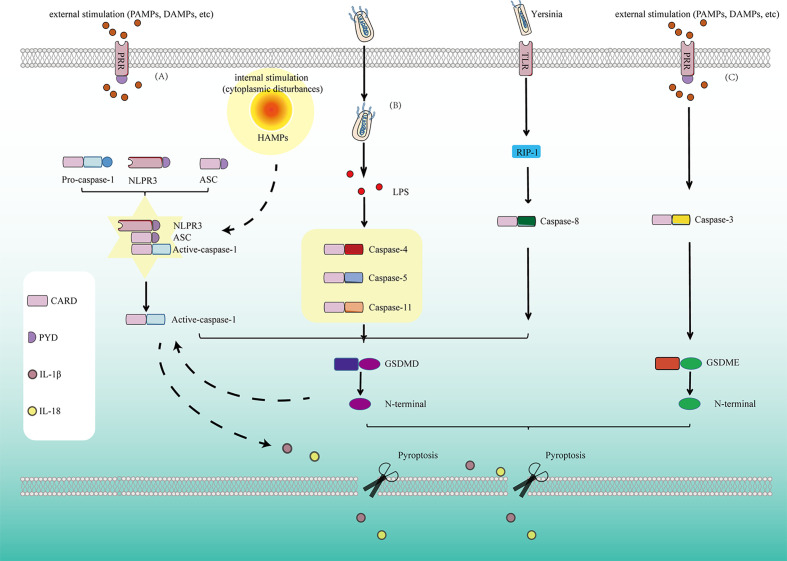
Pyroptosis is mediated by several pathways: **(A)** In the classical pyroptosis signaling pathway, NLRP3 is activated under the stimulation of PAMPs/DAMPs as well as cytoplasmic disturbances, termed as homeostasis altering molecular properties (HAMPs), then the PYD at the NLRP3 N-terminal binds to the ASC protein containing the PYD domain, and the CARD of ASC binds to the CARD of pro-caspase-1 to form a multi-protein complex and activate Caspase-1. Activated Caspase-1 cleaves GSDMD and divides GSDMD protein into lipophilic N-terminal domain and hydrophilic C-terminal domain, the N-terminal domain binds to the lipids on the cell membrane to form pores, causing the release of cell contents. On the other hand, the N-terminal produced by cleavage from GSDMD has another function for promoting the proteolytic activation of caspase-1, helps to form active IL-1β and IL-18. At the same time, cells release IL-1β and IL-18 released to the outside of the cell during pyroptosis recruit more inflammatory cells and expand the inflammatory response. **(B)** In the non-classicial pyroptosis signaling pathway, Caspase-4, 5, and 11 are activated under the stimulation of bacteria and other signals. The GSDMD is cleaved by the activated Caspase-4, 5, and 11 to form the lipophilic N-terminal domain, inducing cell membrane perforation and causing inflammation. **(C)** In Caspase-8-dependent pyroptosis signaling pathway, Caspase-8 is activated by *Yersinia via* the TLR4/RIP1 signaling pathway to induce pyroptosis. **(D)** In Caspase-3-dependent pyroptosis signaling pathway, Caspase-3 promotes the recruitment of the GSDME-N domain to the cell membrane by cutting off GSDME and induces the formation of cell pores, thus leading to pyroptosis.

The caspase-3/GSDME-dependent pyroptosis signal pathway has been discovered in recent years, which is an accessory cell death pathway that occurs when apoptotic cells cannot die efficiently and are cleared (53–55). In recent years, GSDME has been widely studied in addition to GSDMD, it is also one of the effector molecules of pyroptosis. Caspase-3 promotes the recruitment of the GSDME-N domain to the cell membrane by cutting off GSDME and induces the formation of cell pores, thus leading to pyroptosis. Unlike GSDMD-dependent pyroptosis induced by caspase-1, human Caspase-4/5, and mouse Caspase-11, the caspase-3-dependent pyroptosis pathway has been considered a third pyroptosis pathway (56, 57). Therefore, inflammatory caspases ultimately transmit pyroptosis signals to execute GSDMD protein through the classical or non-classical pyroptosis pathway, resulting in pyroptosis (**Figure 1**).

For the third pyroptosis pathway, the distribution and expression level of GSDME determine the mode of pyroptosis mediated by caspase-3 activation. The relatively well-studied GSDM family members are GSDMD and GSDME. Proteolysis of GSDMD forms pores on the membrane. These pores allow the exit of smaller proteins (eg., IL-1β), while the cell remains viable. During this interval which is termed “sublytic phase”, the pores also facilitate uncontrolled osmotic imbalance and eventual disruption of membrane integrity, ultimately leading to the cellular demise in the lytic phase. GSDME can function in either sublytic or lytic phase, Zhou et al. (58) revealed a previously undescribed GSDME sublytic phase during which GSDME pores allow IL-1β release over time, which is similar to GSDMD. The GSDME is found to activate secondarily to IL-1β release in a sublytic phase that is independent of cell lysis, the authors also observed codependency for both GSDMs segregated from their lytic capabilities during IL-1β processing and release, either GSDM activation as a conduit may trigger the activation of downstream, feedforward NLRP3. Therefore, the complex steps of interacted activation and lytic pathways between GSDMD/GSDME deserve further research.

When cells overexpress GSDME, activated caspase-3 will induce pyroptosis. For cells with a low level of GSDME expression, the activation of caspase-3 will trigger the subsequent increase after inducing apoptosis. This caspase-3-dependent mode of cell death is called apoptosis-like cell death. The caspase-GSDME pathway is usually related to the production of intracellular reactive oxygen species (ROS) in cancer cells (59). Several studies have also shown that activating compounds that activate the Caspase3-GSDME pathway is effective in cancer therapy, and GSDME has been considered one of the targets for the treatment of different cancer types (eg, melanoma, lung cancer) (60). For example, the classic anti-tumor drugs cisplatin and paclitaxel, in addition to inducing tumor cell apoptosis, have also been found to participate in the anti-tumor mechanism by inducing pyroptosis through the GSDME pathway (61). Inflammation and tumors are inseparable, and pyroptotic inflammation has different roles in the occurrence and development of tumors, providing new ideas for the treatment of inflammatory diseases and tumors. On the other hand, the caspase-GSDME-pyroptosis pathway is also found related to viral infection. Host cells use additional intracellular sensors called “guard proteins” to dectect pathogenic host-microbe interactions (62). Orzalli et al. (30) found that two disparate viruses (V esicular stomatitis virus and herpes simplex virus-1) resulted in guard protein Mcl-1 depletion and inactivation of guard protein Bcl-xL, leading to caspase-3 dependent cleavage of GSDME and trigger GSDME-dependent pyroptosis. Dong et al. (63) found that enterovirus 71 (EV71) infection induces caspase-3 dependent cleavage of GSDME and resultant pyroptosis, and GSDME deficiency in mice was shown to alleviate pathological symptoms, revealing that GSDME is important for the pathogenesis of EV71 by mediating pyroptosis. At present, the evidence of Caspase-3-dependent pyroptosis induced by virsus is increasing, more research is worthy to explore the role of virus-induced pyroptosis in diseases.

#### 1.1.4 Caspase-8-dependent pyroptosis pathway and other signaling pathways

Apart from the pyroptosis signaling pathway mentioned above, there are other signaling pathways involved in pyroptosis which attracted attention recently. Recent studies revealed an alternative pyroptosis pathway executed by GSDMD during pathogenic *Yersinia* infection driven by a receptor-interacting protein 1 (RIP1)-caspase 8 signaling cascade, which is independent of inflammasomes (64). Caspase-8 was also found able to process IL-1β directly in response to the activation by TLR, death receptor, and dectin-1 pathways (65). GSDME was also found cleaved during pathogenic *Yersinia* infection. Caspase-3/7 cleavage occurred downstream of caspase-8, caspase-8 was found to induce GSDME cleavage mainly through caspase-3 activation with a minor contribution from caspase-7 (64, 66). Consistently, Zheng et al. (67) found that transforming growth factor-β-activated kinase 1 (TAK1) inhibition by pathogenic *Yersinia* infection in macrophages triggers caspase-8 mediated GSDMD cleavage and resultant pyroptosis independent of caspase-1/11, the lysosomal Rag-Ragulator is found necessary for caspase-8 mediated pyroptosis, which may instruct the inflammatory response to *Yersinia*, providing an alternative method of triggering pyroptosis.

Recent studies have suggested alternate proteases to cleave GSDMD, which shed light on potential drug targets for pyroptosis and inflammation (68). It was found that neutrophil-expressed elastase (ELANE) was also able to cleave and activate GSDMD within neutrophils independent of caspases (69). ELANE, a serine protease in cytosolic granules, has been found capable of inducing pyroptosis in neutrophils through cleaving GSDMD and forming pores on plasma membranes. The absence of ELANE or GSDMD was found to increase neutrophils’ lifespan, which suggested ELANE and GSDMD activation could assist in neutrophil death (69). Besides, GSDMD was also found to be activated by Cathepsin G, generating the characteristic N-terminal domain GSDMD-p30 known to induce pyroptosis (70).

In conclusion, key complexes such as inflammasomes, caspases, and GSDMs play an important role in different pathways in pyroptosis. As a topic of a robust field, further research involved in the specific mechanism and diverse signaling pathways of pyroptosis is warranted in the future.

### 1.2 The pathogenic mechanism of pyroptosis in respiratory diseases

The respiratory tract is one of the parts where the human body communicates directly with the external environment, and the innate immune response provides the first line of defense against environmental signals (including pathogens, allergens, and other irritants). Pyroptosis is considered one of the host’s immune defense mechanisms. As a way of regulating cell death, pyroptosis has attracted more and more attention because of its special pathological characteristics (71). The respiratory system could be affected by the pathological factors (LPS, ROS generated by oxidative stress, mitochondrial dysfunction, lysosome released into the cytoplasm, etc) due to the poor local mucosal immunity and the degradation of the surrounding environment (72–74). The pathological factors participate in the activation of NLRP3 inflammasome, which can induce pyroptosis in airway cells (75). Air pollution caused by airborne fine particulate matter (PM2.5) in the air is also one of the main causes of respiratory tract inflammation such as COPD and pulmonary fibrosis. Cao et al. (76) found in 2022 that PM2.5 particles could induce the production of TNF-α and IL-1β in human macrophages and transforming growth factor (TGF) *in vitro*, and could also induce the production of TGF-β1 and IL-1β by activating NLRP3 inflammasome, which could promote pulmonary fibrosis. In brief, emerging evidence indicated that pyroptosis is involved in the inflammatory processes in respiratory diseases (77). Pyroptosis is often accompanied by pathological changes in many kinds of cells. For the respiratory system, pulmonary vascular endothelial cells, bronchial epithelial cells, and alveolar macrophages are vulnerable (78). One possible explanation is that professional phagocytes of these myeloid systems may express higher levels of inflammatory cysteine proteases (caspases-1/11 in mice, and their orthologs caspases-1/4/5 in humans) (10). Excessive pyroptosis may lead to inflammation, tissue injury, and airway damage, and induce an inflammatory reaction, leading to more serious damage and poor prognosis of respiratory diseases, as shown in **Table 1**.

**Table 1:** The pathogenic mechanism of pyroptosis in airway diseases.

Mechanism	Effects	Action	Target	Impact on body
Pyroptosis is mainly mediated by Caspase-1 Caspase-4/5/11, and Caspase-3 signaling pathways, the downstream inflammatory factors IL-1β and IL-18 are released from cells after the activation of GSDMD.	Destruction of cell structure and function	Airway cell injury and inflammation	Pyroptosis of the pulmonary endothelial cells, macrophages, epithelial cells, neutrophils and other cells	Ventilation/perfusion mismatch, severe hypoxemia and poor pulmonary compliance
	Increase the level of TGF- β, stimulate the production of collagen and fibronectin in fibroblasts, and promote the transformation of fibroblasts into myofibroblasts.	Airway epithelial cell repair disorder, pulmonary fibrosis, airway remodeling	Death of pulmonary vascular endothelial cells, recruitment of fibroblasts, and excessive release of ECM	Airflow limitation, airway structural damage and loss of function
	Affect the contraction and relaxation of airway smooth muscle and increase the levels of IgE, IL-1β and Th2 related cytokines	Airway hyperresponsiveness	Airway epithelial cell damage, airway inflammation	Bronchospasm, airway remodeling
	Release of cytokines TNF- α and IL-13	Mucus hypersecretion	Hyperplasia of airway goblet cells and excessive secretion of submucous glandular cells	Airflow limitation and decreased cilia-mucus transport function

**Figure 2:**
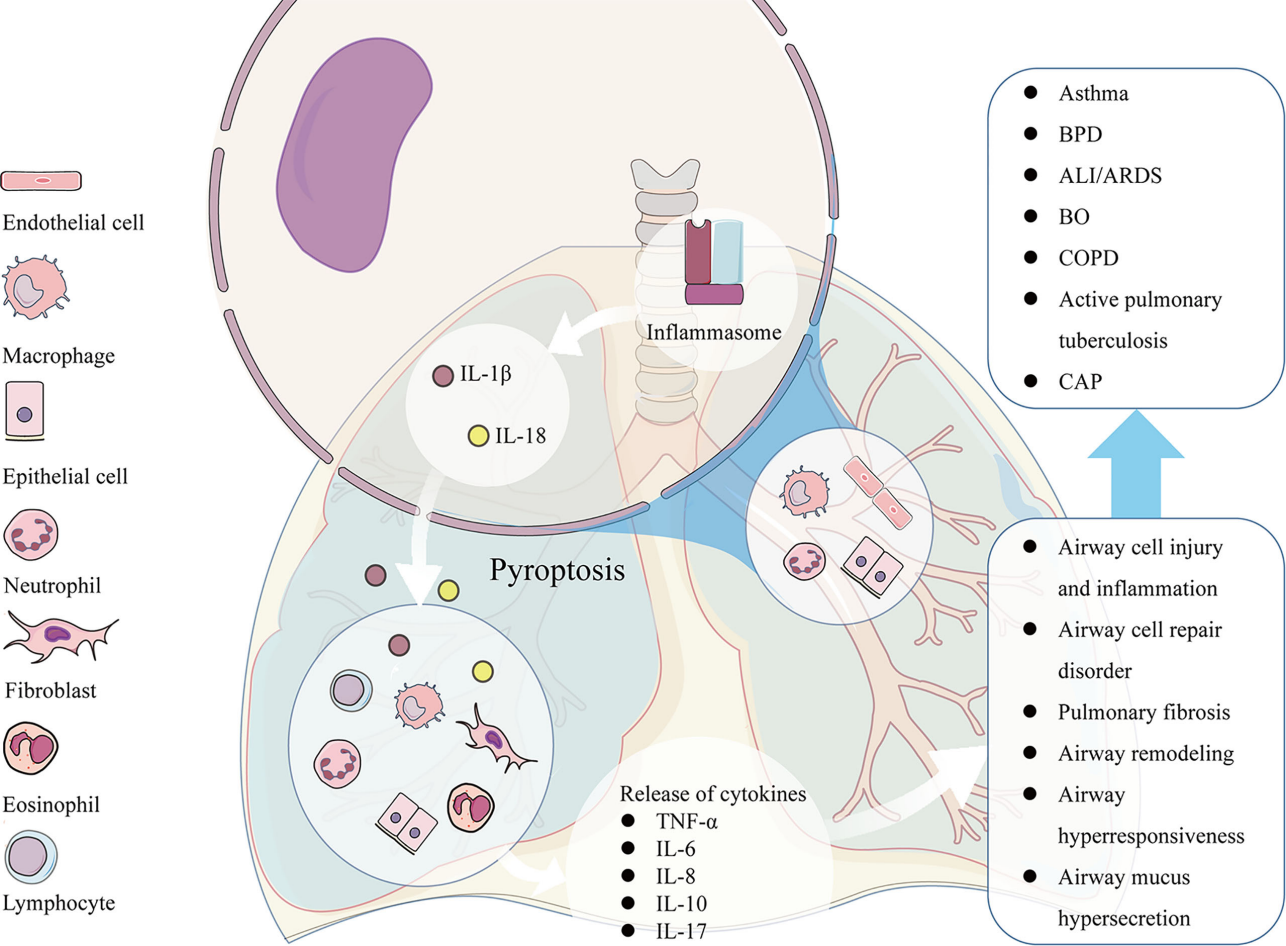
The pathological factors participate in the activation of NLRP3 inducing pyroptosis in many kinds of airway cells, such as pulmonary vascular endothelial cells, bronchial epithelial cells, neutrophils, and alveolar macrophages. The excessive pyroptosis results in the enhanced release of IL-1β and IL-18, which recruit more inflammatory cells and release of other cytokines such as TNF-α, IL-6, IL-8, IL-10, IL-17 and etc., leading to associated pathological consequences, such as airway cell injury and inflammation, airway cell repair disorder, mucus hypersecretion, AHR, and airway remodeling. The pathological changes caused by pyroptosis participates in the pathogenesis of many respiratory diseases such as ARDS, asthma, BO, COPD and so on.

Pyroptosis is an important relationship between innate immune response and pathology of lung diseases. Excessive pyroptosis may lead to airway inflammation, airway injury, airway epithelial cell repair disorder, airway hyperresponsiveness (AHR), and mucus hypersecretion. Pyroptosis participates in the pathogenesis of many respiratory diseases such as acute respiratory distress syndrome (ARDS), bronchial asthma, bronchiolitis obliterans (BO), and so on (**Figure 2**).

## 2 Pyroptosis and inflammation-related respiratory diseases

### 2.1 Severe asthma

Bronchial asthma (referred to as asthma) is the most common chronic airway disease in childhood, characterized by chronic inflammation, AHR, and airflow limitation. Most children respond well to corticosteroid-based treatment in controlling symptoms. However, there are still some children whose asthma symptoms are still not controlled under standardized treatment though they use correct inhalation techniques and have good adherence. Because of the difficulty in treatment, these children also bring heavy mental and economic burdens to the family and society (79). For a long time, asthma has been regarded as heterogeneous chronic lung disease, including several groups of patients with different characteristics or phenotypes. To solve the complexity of severe asthma, predict the prognosis more accurately and apply the corresponding possible targeted therapy, the concept of phenotypes and endotypes of asthma has been widely concerned (80, 81). According to the type of immunocytes response involved in the pathogenesis of the disease, the endotypes of asthma were classified as type 2 asthma and non-type 2 asthma, nearly 50% of people with severe asthma exhibit a T-helper lymphocyte type (Th) 2-mediated, eosinophilic inflammatory endotype, and other subtypes of severe asthma were related to Th1/Th17-mediated response and neutrophil airway inflammation (82–84). In the study of asthma typing in adults, researchers have extensively investigated the types of airway inflammation by using invasive and semi-invasive techniques such as bronchial biopsy, bronchoalveolar lavage (BAL), induced sputum, etc. In sharp contrast to adults, there is a relative lack of such research on asthma in children, and current evidence shows that the phenotypes and endotypes of severe asthma in children may be more complex (81). At present, the pathogenesis of severe asthma is not fully understood, and the development of the treatment of severe asthma is hindered to some extent.

More and more clinical and experimental evidence shows that pyroptosis has special significance in the pathogenesis of severe neutrophil inflammation. NLRP3 and IL-18 protein levels of airway epithelial cells in lung biopsies of patients with asthma were found higher than those of healthy people (85). In addition, the content of IL-1β in serum and the cellular inflammatory factors in sputum and bronchoalveolar lavage fluid of asthma patients also increased compared with healthy people (85–87). Another study showed that the levels of NLRP3 and caspase-1 in the airway of patients with a neutrophilic subtype of asthma were higher than those of healthy people (84). The above results suggest that pyroptosis is involved in the pathogenesis of severe asthma. IL-1β participates in airway inflammatory infiltration, induces AHR, differentiates, and activates Th2 cells, leading to the release of Th2 cytokines (84). In addition, IL-1β related genes are overexpressed in patients with severe asthma, which promotes the differentiation of Th17 and the production of IL-17, thus inducing steroid-resistant neutrophil inflammation (88). An enhanced IL-1β signal was believed a sign of more serious disease (89). These results suggest that pyroptosis is involved in the pathogenesis of severe asthma.

AHR refers to the excessive contractile response of the bronchus to various inhalation stimuli (including chemical and physical stimuli), which is one of the important features of bronchial asthma. In addition to patients with bronchial asthma, AHR can also be detected in patients with rhinitis, smokers, and quitting smokers, after respiratory tract infection and after acute inhalation of irritant chemicals, as well as in airway diseases such as COPD and cystic fibrosis (90). Evidence shows that AHR is related to acute and chronic airway inflammation, respiratory irritation, and airway remodeling caused by chronic inflammation (91). Airway epithelial damage is a sign of respiratory diseases such as asthma. Damaged airway epithelium can induce pyroptosis, which is closely related to the formation of airway inflammation and airway remodeling. Cytokines and chemokines were released to attract inflammatory cells during the pyroptosis progress. IL-1 signal is related to the recruitment of inflammatory cells, especially in eosinophils and neutrophils. IL-1β is one of the IL-1 cytokine families, which affects the contraction and relaxation of airway smooth muscle and plays an important role in AHR. Studies have shown that inhibition of NLRP3 inflammasome can reduce AHR and airway inflammation, accompanied by decreased levels of IL-1β, IgE, and Th2 related cytokines (92). Airway remodeling is a key pathogenic feature of asthma, which is characterized by obvious thickening and structural changes of the airway wall, excessive airway stenosis, and fixed airflow obstruction. It occurs because of persistent inflammatory stimulation, bronchial epithelial injury and airway epithelial cell repair disorders, lung structural destruction, and scar tissue formation. Upon bronchial epithelial injury, activated epithelium secretes growth factors, including IL-13, IL-1β, TGF-β superfamily members, osteopontin and so on, all of which activate the underlying mesenchymal cell and, ultimately lead to basement membrane thickening, subepithelial fibrosis and smooth muscle hyperplasia (93). The recruitment of fibroblasts, the proliferation of these interstitial cells and other interstitial cells that form fibroblast foci, and the release of excessive extracellular matrix (ECM) components, such as fibronectin, collagen, hyaluronic acid, and proteoglycan, are characteristics of fibrosis and one of the important pathogenesis of pulmonary fibrosis and BO (94).

Airway mucus hypersecretion is an important feature of asthma and many chronic airway diseases, which can lead to airflow limitation and a decrease in cilia-mucus transport function (95). Airway mucus is secreted by airway goblet cells and submucous gland cells. Some pro-inflammatory cytokines such as TNF-α can stimulate airway goblet cell proliferation and mucus gene expression (96). More and more evidence shows that the NLRP3 inflammasome is necessary for the activation of allergen-specific Th2 cells. In animal models, NLRP3 inflammasome has also been confirmed to play a key role in allergic asthma (92, 97). Therefore, the activation of the NLRP3 inflammasome can promote the production of TNF-α and IL-13 cytokines and participate in the pathogenesis of airway mucus hypersecretion.

The activation of inflammasomes may play an important role in the pathogenesis of asthma. Studies have demonstrated that NLRP3 inflammasome activation is involved in the allergen-driven airway inflammatory responses in mouse models of allergic asthma (71). Besides, increased mRNA expression of inflammasome sensors (NLRP1, NLRP3, and NLRC4) and increased cytokine IL-1β levels are observed in the sputum samples of patients with neutrophilic severe asthma (71, 98, 99). At present, preventing the activation of NLRP3 inflammasome has become a hot spot in the treatment of severe asthma (71). MCC950 is one of the most studied direct inhibitors of the NLRP3 pathway at present, it directly and specifically binds to NLRP3 irrespective of its activation state which occurs at or near the Walker B site of NLRP3, blocking the ATP hydrolysis required for NLRP3 inflammasome function (100). MCC950 was found to prevent the development of the pathology and reduce the expression of inflammatory mediators, such as IL-1, TH2-derived cytokines, etc. in severe asthma mice model induced by house dust mite allergen and Complete Freund’s Adjuvant (101). Toluene Diisocyanate (TDI) is a known respiratory sensitizer linked to occupational asthma, MCC950 has also shown efficacy in a TID-induced asthma model, in which it was found to inhibit NLRP3 activation and reduce downstream inflammatory mediators (such as caspase-1, IL-1β, and IL-18) levels, alleviating AHR, airway inflammation, and airway remodeling (102). Efficacy with MCC950 has also been shown in an OVA-sensitized model in which it was found to reduce IL-1β and TH2 cytokine levels (100). Except for MCC950, multiple drugs, such as 3,4-methylenedioxy-β-nitrostyrene, bioactive derivatives (eg, echinatin, etc.) from medicinal plants, have been found to interfere directly with the NLRP3 (91, 103). NLRP3-targeted therapies provide potential and prospects for the treatment of related diseases including severe asthma. Other indirect inhibitors of the NLRP3 pathway were also found potential. The production of ROS is important for the activation of NLRP3 inflammatory vesicles because ROS scavenger (NAC) blocks the release of inflammatory cytokines and the activation of NLRP3 inflammatory vesicles. Kim et al. (87) found that ovalbumin activates the NLRP3 inflammasome by inducing the production of mitochondrial ROS, and the levels of Caspase-1 and IL-1β are significantly increased. Under the processing of mitochondrial ROS inhibitor NecroX-5, the activation of NLRP3 inflammasome was inhibited, resulting in a decrease in the level of IL-1β in cells. Peroxisome proliferator-activated receptor γ (PPAR-γ) also plays an important role in the regulation of inflammation. PPAR-γ receptor agonist rosiglitazone was found to inhibit the expression of NF-kB in asthmatic mice, reducing the expression of downstream inflammatory factors IL-4 and IL-13, and further inhibiting the activation of TLR2/NLRP3 inflammasomes, which ultimately inhibited the pyroptosis-related signal transduction pathway and reduced airway inflammation in asthmatic mice (104). Pinkerton et al. (105) recently found that depletion of Th2 cytokines (IL-5 and IL-13) inhibited NLRP3 inflammasome responses, and reduced steroid-insensitive AHR in experimental obesity-induced asthma, however, inhibition of NLRP3 inflammasome had no effect on IL-5 or IL-13 responses in experimental asthma, which highlighted the potential utility of T2 cytokine-targeted biologics.

Strategies for asthma therapy targeting other components of the pyroptosis pathways also deserve attention. Caspase-1-deficient mice model showed significantly reduced eosinophils, and Th2 upstream cytokines (such as IL-33 and IL-25) were reduced during exacerbations. Furthermore, rhinovirus infection was found to increase the level of caspase-1 in asthmatic patients’ bronchial epithelial. Caspase-1 induces Th2 upstream cytokines and eosinophilia in the lung during exacerbations (106). Besides, caspase-11-deficient mice were also found resistant to developing experimental allergic airway inflammation, suggesting that non-classical pyroptosis pathway may be involved in allergic airway inflammation (107). Zasłona et al. (107) observed a dramatic increase in caspase-11 expression in the ovalbumin mouse model of allergic airway inflammation in their experiment, Caspase-11-deficient mice were shown resistant to developing experimental allergic airway inflammation, where Prostaglandin E2 (PGE2) is shown to be protective through inhibiting caspase-11-dependent pyroptosis in murine and human macrophages. Finally, in alveolar macrophages, they observed an increased expression of caspase-4, which is a human homologue of mouse caspase-11, suggesting that human caspase-4 and mouse caspase-11 contribute to allergic airway inflammation and are involved in the pathophysiology of asthma (107). Therefore, inhibition of caspase-1/4 may be a promising therapeutic target in asthma.

Additionally, other members of the GSDM family which act as pyroptotic executioners were found related to the pathogenesis of asthma (17). The interplay between DNA methylation and GSDMA expression was found related to an individual’s susceptibility to asthma (108). However, the relationship between GSDMA-induced pyroptosis and asthma remains unclear. GSDMB was found highly expressed in lung bronchial epithelium in asthmatics (109). Markers near the ORMDL3/GSDMB genes on chromosome 17q21 were found strongly associated with childhood-onset asthma, among which the rs7216389 was the most strongly associated with disease (110). A meta-analysis in 2018 found significant association between GSDMB rs7216389 and pediatric asthma risk (111). Ober et al. revealed that 17q12-21 single nucleotide polymorphisms regulating GSDMB expression in airway epithelial cells, but not in peripheral blood mononculear cells, play a major role in childhood-onset asthma based on genetic and gene expression data in African Americans and European Americans, which indicated that GSDMB may be the leading candidate gene at the asthma locus (112). In addition, recent research revealed that the increased level of GSDMB contributed to inducing an asthma phenotype characterized by AHR and airway remodeling without lung inflammation (109). The upregulated expression of GSDMB in bronchial epithelial cells was also found to increase the susceptibility to asthma (113). It was also revealed that some GSDMB-regulated genes, cytokines, and chemokines, such as TGF-β1, MMP-9, cysteinyl leukotrienes (LTC4/D4/E4), CXCLs, and so on, contribute to inflammation and airway remodeling in asthma (109, 112, 114, 115). These results indicate that GSDMB-induced pyroptosis is involved in the pathogenesis of asthma, nonetheless, more comprehensive studies are needed to identify the gene-gene and gene-environment interactions with respect to asthma.

Therefore, the inhibition of pyroptosis may be beneficial to AHR and Th2 inflammation. However, some studies suggest that the role of NLRP3 inflammasome activation in allergic airway inflammation is debatable. For example, in 2012, Allen et al. (116) found that in NLRP3(-/-), Caspase-1(-/-), or PYCARD(-/-) mice, aluminum hydroxide was not affected as an adjuvant in the model of allergic airway inflammation induced by adjuvant-free Ovalbumin (OVA). A role of NLRP3 inflammasome in the development of allergic airway disease induced by acute or chronic exposure to house dust mite antigens was also not detected. Another study found that NLRP3 (-/-) mice had no significant difference in airway eosinophil proliferation, mucus production, AHR, and Th2 cell responses when exposed to uric acid crystals or particulate matter (PM)/OVA-induced experimental asthma mice (117). Nonetheless, some controversial results obtained in animal studies may be mainly related to changes in the experimental models used, including the type and concentration of allergens, the route and time of administration, and differences in mouse strains, notably, these animal models mentioned above are mainly focused on Th2 asthma. Therefore, it is necessary to carry out further mechanism research to solve these differences.

Although the exact role of pyroptosis activation in the experimental animal models of asthma is not completely clear, the key complexes such as NLRP3, GSDMB, GSDME, etc., and related signaling pyroptosis pathways are still promising therapeutic strategies. It is worth further studying the role in various asthma subtypes and endotypes.

### 2.2 Pulmonary fibrosis (asbestosis, silicosis, and idiopathic pulmonary fibrosis)

Pulmonary fibrosis is a deadly disorder characterized by a collection of ECM in interstitial tissue and basement membranes (94). There are respirable particles such as crystalline silica dust in many industries and occupations, studies have shown that the damage and persistent damage of residual persistent particles to pulmonary epithelial cells are driven by the inflammatory process, leading to lung diseases elicited by environmental exposures (eg, asbestosis and silicosis) (118). Idiopathic pulmonary fibrosis (IPF) is a chronic and irreversible lung disease with not fully understood etiology and limited therapeutic strategies, novel therapeutic approaches are clinically needed. Inflammation plays an important role in the occurrence or development of fibrosis. In the process of lung injury, necrotic pulmonary vascular endothelial cells can recruit macrophages, monocytes, and other inflammatory cells, causing them to secrete IL-18 and IL-1β, and further induce pulmonary endothelial cell death, peripheral tissue inflammation, and pulmonary fibrosis progression (118–120). It is known that IL-1β can promote the production of fibrogenic cytokine TGF-β, increase the level of TGF- β, and stimulate the production of collagen and fibronectin in fibroblasts, thus promoting the transformation of fibroblasts to myofibroblasts (121). IL-18 is also thought to be involved in the development of fibrosis (122). Caspase-1 helps to form active IL-1β and IL-18, involved in fibrogenesis, in addition, recent evidence also suggests that inflammasomes, particularly the NLRP3 inflammasome, might be involved in the pathogenesis of fibrosis (123).

Vitro studies have demonstrated that pyroptosis is triggered in silica-treated alveolar macrophages through the classical NLRP3/caspase-1 pyroptosis pathway (124). It was found that ROS generation induced by silica accelerated pyroptosis in macrophages *via* the NLRP3/caspase-1 signaling pathway, blocking macrophages’ pyroptosis by inhibiting the NLRP3 inflammasome or caspase-1 could reduce silica-mediated pulmonary fibrosis (125). Additionally, it is revealed that caspase-11 was upregulated in mice exposed to silica, caspase-11 inhibition reduced GSDMD-induced pyroptosis in silica-treated macrophages, yet the specific mechanism was unclear (124). NLRP3 inflammasome was also found overactivated in IPF patients, causing increased production of IL-1 cytokine families and collagens (126). NLRP3 inhibitors have been developed to interfere with the NLRP3 machinery, and NLRP3-knockout has been investigated in different animal models of pulmonary fibrosis. NLRP3 inhibitor MCC950 was found to block IL-1β in mouse lungs with cystic fibrosis, which reduced airway inflammation (127). Recently, Liang and his colleagues (128) found that an alkaloid “Lycorine” which is isolated from Amaryllidaceae family plants, ameliorated bleomycin-induced pulmonary fibrosis by inhibiting NLRP3 inflammasome activation and pyroptosis *via* targeting the PYD domain of ASC. In addition, Drosha ribonuclease III, a class 2 ribonuclease III enzyme, was found to promote AIM2 inflammasome activation-dependent lung inflammation during IPF (129). In brief, inflammasomes (eg., NLRP3 inflammasome) involve in the pathophysiology of fibrotic lung diseases, studies suggest that NLRP3 inhibition could provide a promising approach against the fibrotic pulmonary process. Although multiple NLRP3 inhibitors are available, their proper use and the function of other inflammasomes still deserve further research.

### 2.3 Bronchopulmonary dysplasia

Newborns and premature infants are particularly vulnerable to infection and inflammation. Bronchopulmonary dysplasia (BPD) is a common pulmonary complication in premature infants, which is mainly seen in premature infants. The etiology is related to many factors (130). Intrauterine infection and postnatal infection, hyperoxia-induced pulmonary dysplasia, alveolar damage, and abnormal vascularization are the key factors in the pathogenesis of BPD, which not only lead to neonatal chronic lung diseases but also leave long-term pulmonary dysfunction, seriously threatening the physical and mental health of children and causing heavy mental and economic burden to the society (131, 132). There is no specific treatment for BDP. The mortality and complication rates of premature infants with BPD are significantly higher than those of general premature infants (131).

Recent studies have shown that the pathogenesis of BPD is associated with inflammasomes. It was observed in the animal models that the expression of NLRP3, TNF- α, IL-1 β, and IL-6 in the lung tissue of BPD neonatal mice was significantly increased, the inflammatory infiltration of the lung tissue was obvious, the alveolar septum thickened, the alveolar structure and the number of alveoli decreased (133). Oxidative stress injury plays an important role in the pathogenesis of BPD. Dapaah-Siakwan (134) and his colleagues found that hyperoxia could activate NLRP1 inflammasome, increase the production of mature IL-1β and upregulate the expression of p30 GSDMD, while the application of Caspase-1 inhibitor Ac-YVAD-CMK could attenuate the activation of NLRP1 inflammasome induced by hyperoxia, inhibit subsequent cell death mediated by pyroptosis in the lung, reducing IL-1 β activation and p30 GSDMD expression, improving alveolar and vascular development in hyperoxia-exposed lungs, and alleviating lung and brain injury induced by hyperoxia in neonatal mice. Liao et al. (135) found the activation of NLRP3 inflammasome in hyperoxia-induced BPD neonatal mice at 85% hyperoxia, resulting in increased IL-1β and abnormal alveolar formation, while the use of recombinant IL-1RA (blocking the interaction between IL-1 and its receptor) and glibenclamide (a compound that blocks the assembly of NLRP3 inflammasome) showed the effect of blocking inflammasomes and protecting lung structure compared with placebo-treated mice. These accumulating results suggest that inflammasomes and their associated inflammatory proteins play a key role in the pathogenesis of BPD and represent a potential therapeutic target for the treatment of this devastating infant disease.

### 2.4 ALI/ARDS

ALI is a life-threatening disease syndrome, which is often the main cause of ARDS. ARDS is a clinical syndrome characterized by injury of pulmonary capillary endothelial cells and epithelial cells, which is the ultimate common result of lung injury caused by many causes, persistent damage to airway epithelium and chronic inflammation are also markers of the pathogenesis of chronic airway disease (136, 137). The lung tissue of ARDS patients shows a severe inflammatory reaction, so it is one of the necessary measures for prevention and treatment to control the primary disease and curb the uncontrolled inflammatory reaction of the whole body (138). Recent studies have shown that pyroptosis is associated with the pathogenesis of ALI/ARDS (139).

The precipitating factors of ALI/ARDS are legion, and include, but are not limited to severe shock, infection, mechanical injury, etc. (140, 141). Over the years, a large number of studies have demonstrated that gram-negative bacterial infection is one of the most important factors leading to ALI (141). The pulmonary endothelial cells are often exposed to LPS or other pathogens circulating in the blood, they account for nearly 50% of the total number of lung cells, regulating vascular tension, permeability, ventilation-perfusion matching, and interaction with blood-derived cells (142). Pulmonary endothelial cell injury and death occur throughout all stages of ALI/ARDS (143). LPS is a component of the outer wall of Gram-negative bacteria, which is reflected by TLR4 on the cell membrane surface of host cells. LPS and hemorrhagic shock (HS) can induce endothelial cell pyroptosis through the NLRP3/caspase-1/GSDMD classical signal pathway (144). It was demonstrated that LPS could upregulate the expression of caspase-11 in mouse endothelial cells *via* TLR4 and then induce pyroptosis of endothelial cells through NLRP3/caspase-11/GSDMD pathway, besides, conditional deletion of caspase-11 in endothelial cells was found to reduce endotoxemia-induced lung edema and death, which indicated an essential role for caspase-11 in endotoxemia-induced ALI (139). HS promotes the formation of inflammasomes through the HMGB1/RAGE signal pathway, lytic caspase-1 can activate downstream pathways caspase-3 and caspase-9, and activate the caspase-3-mediated pyroptosis pathway. In addition, Homocysteine and LPS play a synergistic role in endothelial pyroptosis (145). Therefore, endothelial inflammatory caspases may be an potential therapeutic target for ALI/ARDS.

Macrophages are innate immunity and necessary in host defense. By releasing inflammatory cytokines and regulating other immune cells, macrophages are indispensable in the initiation and regression of pulmonary inflammation (146). Macrophage pyroptosis mediated by NLRP3 induces the secretion of a nuclear protein high mobility group box 1 (HMGB1) by macrophages from ALI patients, which is one of the key molecules of the IL-17 signaling pathway. HMGB1 as a DAMP can promote the activation of NLRP3 and caspase-1, enhancing the pyroptosis, and aggravating the degree of ALI through positive feedback (147). In addition to Gram-negative bacteria, Gram-positive bacteria also have the mechanism of activating NLRP3. For example, *Streptococcus pneumoniae* can disrupt the plasma membrane by producing cytokines, aerolysin, and other toxins, leading to K + outflow and activating the NLRP3 inflammasome in macrophages (148). Except for NLRP3, studies also showed that other inflammasomes were related to the pathogenesis of ALI. It was revealed that anthrax lethal toxin could induce NLRP1-dependent pyroptosis in mouse macrophages, which promoted lung inflammation and resultant exacerbation of ALI (149). Recently, Li et al. found that the activation of NLRC4 inflammasome in macrophages triggered by *pseudomonas aeruginosa* infection led to pulmonary inflammatory damage, while Adipose-Derived Mesenchymal Stem Cells improved lung tissue damage in mice through reducing the NLRC4 inflammasome activation in macrophages (150). Wang et al. found that HMGB1 could participate in LPS−induced of ALI by activating the AIM2 inflammasome in macrophages (151). Furthermore, growing evidence suggests that pyroptosis may also enhance the inflammatory response in ALI by generating pyroptotic bodies (PyrBDs) which are released by macrophages in a Caspase-1-dependent manner. PyrBDs serve as mediators of LPS-induced ALI by triggering vascular interstitial edema and recruiting neutrophils (152). In addition to bacteria, viruses can also induce ALI, and some severe cases have serious complications, such as systemic inflammatory response characterized by the release of inflammatory cytokines, ARDS, respiratory failure, and so on (153). High levels of IL-1β were detected in bronchoalveolar lavage fluid and blood plasma of patients with lung injury caused by coronavirus or other respiratory viruses (such as adenovirus or influenza virus) infections. With the widespread coronavirus disease (COVID-19) in 2019, the infection has spread all over the world. Respiratory failure can occur in severe cases, which can be accompanied by a systemic inflammatory response characterized by the release of inflammatory cytokines. It has been found that the injury of type II alveolar epithelial cells expressed by angiotensin-converting enzyme 2 (ACE2) leads to the activation of the NLRP3 inflammasome. The acute immune response to Severe acute respiratory syndrome coronavirus-2 (SARS-CoV-2) infection is largely driven by inflammatory alveoli and monocyte-derived macrophages, which are activated by PAMPs and DAMPs and released by infected and apoptotic lung cells. The mRNA of tumor necrosis factor-α (TNF-α) and IL-1β secreted by alveolar macrophages initiated an acute pro-inflammatory cascade immediately after infection, which could lead to a storm of inflammatory cytokines, suggesting that pyroptosis-related cells were involved in the systemic inflammatory response of severe COVID-19 (153). Another mechanism of activating NLRP3 inflammasome is related to direct interactions with viral proteins. Another mechanism of activating NLRP3 inflammasome is related to direct interactions with viral proteins. The SARS-CoV protein ORF8b can directly activate the NLRP3 inflammasome by binding the leucine-rich repeat region of NLRP3 or interacting with the inflammasome adaptor ASC (154). Additionally, SARS-CoV-2 infection in blood monocytes was found to activate NLRP3 and AIM2 inflammasomes, leading to pyroptosis and inflammation (155). Recently, human caspase-4 and its mouse homolog caspase-11 were found up-regulated in SARS–CoV-2 infections, in which CASP4 expression was demonstrated to correlate with the severity of human SARS–CoV-2 infection, however, SARS–CoV-2–induced inflammation triggered by caspase-4/11 were largely independently of GSDMD (156). Additionally, Li et al. (157) found that SARS-CoV-2 triggered inflammatory responses and cell death (mainly apoptosis) through caspase-8 activation, it was noted that the necroptosis inhibitor did not fully block IL-1β secretion during SARS-CoV-2 infection, which suggested that caspase-8 dependent pyroptosis might be involved in the SARS-CoV-2-induced inflammatory responses. Therefore, key compotents (such as NLRP3 inflammasome, caspase-1,-4,-8,-11 and so on) in signaling pathways associated with pyroptosis might be promising drug targets for severe COVID-19 treatment.

Pyroptosis of pulmonary epithelial cells, neutrophils, and other cells has also been found to be involved in the occurrence of ALI, and ultimately affect the development and prognosis of ALI (147). In the process of pyroptosis of airway cells including airway macrophages, neutrophils, and endothelial cells induced by inflammatory caspase, a large number of studies have shown that NLRP3 inflammasome mediators and IL-1β mediate the inflammatory response in ALI and ARDS. The levels of IL-1β in bronchoalveolar fluid and plasma in patients with ARDS were higher than those in healthy controls and were associated with poor clinical outcomes. The inflammasomes especially NLRP3, are important regulatory targets and play an important role in pyroptosis (142). In addition to LPS and virus, other causes of ALI include ventilator-induced lung injury, transfusion-related acute lung injury, and so on, the mechanisms are related to the inflammatory response caused by IL-1β and IL-18 induced by pyroptosis (158).

Strategies for ALI/ARDS therapy targeting pyroptosis pathways deserve attention. Caspase-1 inhibitors have shown beneficial efficacy in ALI/ARDS in animal models. For example, in the LPS-induced mouse ALI/ARDS model, the caspase-1 specific inhibitor Ac-YVAD-CMK can inhibit the pyroptosis of macrophages and the release of inflammatory factors in the alveoli (159). Caspase-1 mediates the expansion of intrinsic lymphoid cells 2 (ILC2) in the lung through its receptor ST2, IL-33 was found to inhibit the function of caspase-1 in endothelial cells (160). Besides, it was found that tetramethylpyrazine could alleviate ALI by suppressing the TLR4/TRAF6/NFκB/NLRP3/caspase-1 and TLR4/caspase-8/caspase-3 signaling pathways (161). Therefore, further study on the relationship between pyroptosis and pulmonary vascular endothelial cells can increase researchers’ understanding of ALI/ARDS and provide help for diagnosis and treatment.

### 2.5 BO

BO refers to the inflammation and immune reaction that damage bronchiolar epithelium and subepithelial tissue, leading to pathological changes due to the imbalance of the tissue repair process. BO is a pathological concept, characterized by inflammatory cells infiltration around bronchioles, which eventually damages the airway, followed by fibrous hyperplasia, granulation tissue formation, and the accumulation of extracellular matrix, resulting in airway fiber occlusion and decreasing the quality of life of patients (162). BO is considered to be divided into three entities: post-infected BO (PIBO), BO after allogeneic lung transplantation, and BO after allogeneic bone marrow transplantation (BMT) or hematopoietic stem cell transplantation (HSCT) (163). Infection is the first cause of BO in children. The most common pathogen of PIBO is adenovirus (ADV) especially serotypes 3, 7, and 21. There are also reports that PIBO is secondary to influenza and other viruses (164). The development of BO involves a variety of mechanisms. More and more clinical and experimental evidence shows that NLRP3 inflammasome activation and its induced pyroptotic response have special significance in the pathogenesis of BO (163). Some pathogens (such as ADV, and influenza) can induce pyroptosis by activating the NLRP3 inflammasome, releasing IL-1β, and causing lung injury, and the process of tissue repair is maladjusted, which is related to the pathogenesis of PIBO. In addition, the activation of NLRP3 inflammasome is also involved in other BO entities (165). ADVs are recognized as important pathogens of respiratory tract infections in children and are divided into multiple serotypes, especially adenovirus types 3, 7, and 21, which may cause high morbidity and mortality in children with pneumonia, and are closely associated with PIBO in children, which is a hot spot in children’s PIBO research (162). Studies have shown that ADV (types 3 and 7) infection of host respiratory epithelial cells stimulates the innate immune system to produce different inflammatory responses, which may partially explain the different severity of lung inflammation caused by some ADV types. Airway epithelial cells are not only passive barriers to infectious particles but may also be involved in innate immune responses (166). The initial inflammation of ADV infection is neutrophil interstitial infiltration with neutrophil alveolitis. Monocyte infiltration became obvious over time, and monocyte-derived macrophages produce IL-1β through pyroptosis. The mechanism may be that ADV activates Caspase-1 and NLRP3 inflammasome through the HMBG1 pathway, and can also activate the complement system to produce allergic toxin C3a, activate Caspase-1, and cause ALI through the classical pyroptosis pathway (167).

It has been shown that activation of the NLRP3 inflammasome, an important component of innate immunity, is significantly elevated in mice receiving allogeneic tracheal grafts (168). Acquired immunity is also involved in the pathogenesis of BO. Helper T cell 1 (Th1) and helper T cell 17 (Th17) play a key role in mediating allograft rejection. IL-1β and IL-18 can cooperate with IL-23 to promote the production of Th17 cells and IL-17, promote Th1/Th17 response and inhibit Treg response, and participate in the pathogenesis of BO after transplantation (169). Therefore, pyroptosis is closely related to the occurrence and development of BO.

The treatment options for BO are limited, and there is no specific treatment at present. Although some medicine such as immunosuppressants and macrolides are increasingly used in the treatment of BO, their curative effects on BO are still not completely clear, and the possible drug side effects need to be clinically noted (170). Therefore, a new treatment is still needed to improve the prognosis of BO. In 2020, D’Amico et al. (171) found that the formyl peptide receptor 1 gene deletion has a protective effect on BO induced by heterotopic tracheal transplantation in mice by regulating the signal transduction of NLRP3 inflammasome and reducing its activation. In 2020, Xu et al. (168) found that in the orthotopic tracheal transplantation mouse BO model, NLRP3 inflammasome inhibitor MCC950 blocked the activation of NLRP3 inflammasome to reduce the production of pyroptosis-related cytokines IL-1β and IL-18, thus regulating the balance of Th1/Th17 and Treg cells and improving BO lesions. Therefore, inhibiting the activation of NLRP3 inflammasome and inhibiting pyroptosis may be a new target for the treatment of BO. Although the relationship between BO (especially PIBO) and pyroptosis remains a limited field at present, further study is deserved to explore the role of pyroptotic key complexes (such as inflammasomes) in the pathogenesis of PIBO and post-transplant BO.

### 2.6 COPD

COPD includes the clinical phenotype of chronic bronchitis and airway obstruction exacerbated by secondary infection, as well as emphysema. As a devastating lung disease, COPD is characterized by airflow limitation that is not fully reversible, airflow limitation is usually progressive and associated with an abnormal inflammatory response of the lungs to noxious particles or gases.

Cigarette smoke is a major risk factor for COPD. Targeting lung epithelial cells, cigarette smoke can activate other lung cells, including inflammatory cells. In COPD patients, NLRP3 inflammasome is over-expressed in the lung and the expression is related to airflow obstruction (172). At different stages of COPD, Faner et al (172) found in 2016 that the miRNA content of NLRP3 and IL-1β increased in stable COPD patients, but Caspase-1 and ASC were not activated. Compared with clinical convalescent patients, the contents of Caspase-1, ASC, and downstream cytokines IL-18 and IL-1β in patients with severe disease were increased. Wang et al. (173) found that the expression levels of NLRP3 inflammasome in peripheral blood mononuclear cells (PBMCs) and bronchial tissues from patients with acute exacerbation of COPD were significantly higher compared to those in smokers without lung diseases. Studies have shown that elevated levels of IL-18 and IL-1β, which are cytokines of downstream effector molecules in the NLRP3 inflammatory pathway, have been detected in the lungs of mice exposed to cigarette smoke and in human patients with COPD (174, 175). The expression of IL-18 induced inflammation similar to COPD lesions in mature murine lungs (175). The level of IL-1β was found to increase in sputum and serum of COPD patients, and the serum IL-1β level correlated with disease severity (174). Additionally, human caspase-4 and mouse caspase-11 may contribute to airway inflammation in COPD. It was found that in COPD patients, PBMCs treated with combustion-generated ultrafine particles (UFPs) released caspase-4-dependent inflammasome compared with healthy subjects, besides, IL-18 and IL-33 released from PBMCs of unstable COPD patients were found correlated to caspase-4 release, instead of caspase-1 or caspase-8-dependent (176). AIM2 inflammasome expression was found higher in lung recruited dendritic cells and macrophages in smoking mice, which was related to the activation of caspase-11, rather than caspase-1 (177).

As discussed previously, cigarette smoke is the main cause of COPD, while other factors such as air pollution, infection, occupational chemicals and dust, and so on, may also be involved in the pathogenesis of COPD (178). Studies revealed that PM2.5 enhances morbidity and mortality in COPD (178). PM2.5 was found to promote pulmonary fibrosis by activating the NLRP3 inflammasome (76). Occupational dust such as silica dust increases the risk of COPD, the mechanism may be the occupational dust active the NLRP3 inflammasome (118). Besides, studies provided evidence that pathogens such as human rhinovirus, and influenza may contribute to COPD exacerbation by activating the NLRP3 inflammasome (178). Future studies investigating the role of NLRP3 inflammasomes and other signaling pathways during COPD progression may contribute to the disease therapy.

Inhibition of the pyroptosis pathway is a hot topic in the prevention and treatment of COPD. It has been found that down-regulation of caspase-1 expression and inhibition of NLRP3 inflammasome can reduce experimental COPD in mice. NLRP3 inhibitor MCC950 has been shown to prevent LPS-induced pulmonary inflammation in mice (179). However, in clinical application, randomized clinical trials have shown that indirect inflammasome inhibitors such as IL-18 and IL-1β monoclonal antibodies have no effect in patients with moderate to severe COPD, indicating that although IL-1-like cytokines exist during exacerbations of the COPD, other mechanisms are involved in the pathogenesis of COPD and may be the main cause of inflammatory activation.

## 3 Pyroptosis and infection-related respiratory diseases

### 3.1 Active pulmonary tuberculosis

The essence of pulmonary tuberculosis is a bacterial infection. The pathogen *Mycobacterium tuberculosis* (Mtb) spreads through the air and mainly affects the lungs, worse still, it can spread to other organs and cause active tuberculosis, which may lead to inflammatory symptoms of spreading foci. At present, the incidence of tuberculosis has rebounded in many countries, and the high prevalence of tuberculosis worldwide is partly due to the development of drug-resistant strains, which makes the current treatment tricky (180). When the body is infected with Mtb, it can produce cell-mediated immune responses and allergic reactions that may lead to the spread of tuberculosis. Studies have shown that excessive inflammation can increase the severity and complications of active tuberculosis (181). Therefore, regulating the immune system during Mycobacterium tuberculosis infection may be a promising strategy for controlling the mycobacterial infection.

Innate immune response and proinflammatory cytokines also play an important role in active pulmonary tuberculosis. The activation of NLRP3 inflammasome in alveolar macrophages and pulmonary epithelial cells is an important innate immune response to Mtb, promoting the production of the IL-1 cytokines family (182, 183). Studies have shown that the activation of inflammasomes and the early production of IL-1 cytokines family contribute to the early immune response of Mtb, thus providing a potential control strategy (184). However, Mtb can also excessively activate inflammasome, induce cell death, cause immunopathological damage, and enhance tissue damage, which is related to the severity of the disease (181, 185). The mechanism of inflammasomes activity caused by Mtb is not completely clear, but early studies have found that it is related to some proteins secreted by Mtb, which can activate caspase-1 to induce the classical pyroptosis pathway (186). In recent years, it has also been found that it may be related to the interaction between cell surface Mtb antigen and TLR4 (187). Cytokines such as chemokine IL-8 (CXCL-8) are often detected in patients with tuberculosis, and their role in active tuberculosis has been paid more and more attention (188). Most subjects of the current studies are adults. In 2016, Lui et al. (181) found that pro-inflammatory cytokines IL-8 (CXCL-8) and IL-18 were significantly increased in patients with active pulmonary tuberculosis, and the plasma levels of these proinflammatory cytokines (such as IL-8/CXCL-8, IL-18) were significantly correlated with mycobacterial load, degree of X-ray consolidation, tuberculosis severity score, and length of hospitalization. The mechanism may be the existence of the HMGB1/RAGE signal pathway to promote the formation of inflammasomes, inducing pyroptosis.

Recent studies have also explored that some medicinal plants and their bioactive derivatives (such as Andrographolide, Baicalin, Micheliolide, GuttiferoneK, etc.) may reduce the production of IL-1β and IL-18 cytokines by inhibiting the pyroptosis induced by Mtb infection, inhibiting the NLRP3 inflammasome-related pathway and finally reducing the production of IL-1β and IL-18 cytokines. The mechanism is that these medicinal plants and their active derivatives can inhibit NLRP3 inflammasome in Mtb-infection models at transcriptional and post-transcriptional levels, so they can be used as host-directed adjuvants to control excessive inflammation induced by Mycobacterium tuberculosis. However, due to the lack of experience in the application of these drugs in children, more studies are needed to explore the specific role of many NLRP3 inflammasome regulatory factors in Mtb-infection models *in vitro* and *in vivo*, provide a new perspective on the control of immunopathology of pulmonary tuberculosis.

### 3.2 Community-acquired pneumonia

CAP is a common infectious disease, infection may be caused by viruses, bacteria, or fungi. CAP is characterized by inflammation caused by damage to pathogens and the detection of infection in the immune system (189, 190). In 2019, COVID-19 caused by SARS-CoV-2 has led to a high incidence in the world, which has aroused widespread concern all over the world (191). COVID-19 usually presents as mild cold-like symptoms, but some cases may be accompanied by serious and life-threatening complications such as ARDS. Tissue damage is not the result of viral replication or infection, but rather the dysregulation of an excessive inflammatory response to viral infection. The pathology is characterized by intense and rapid stimulation of the innate immune response, activation of NLRP3, induction of excessive pyroptosis, and release of pro-inflammatory factors such as IL-1β (153). In summary, COVID-19 has resulted in a range of disease manifestations, the most severe of which is mediated by a massive inflammatory response that stimulates the NLRP3 inflammasome. Interestingly, epidemiological studies have always shown that children infected with SARS-CoV-2 have a mild clinical course and significantly lower morbidity and mortality than adults, which is in contrast to other respiratory viruses, such as influenza virus, respiratory syncytial virus, and ADV. Children are usually more severe than adults in respiratory diseases caused by these viruses (192, 193). A recent study showed that the expression of SARS-CoV-2 copy, angiotensin-converting enzyme 2, and TMPRSS2 genes in children was similar to that in adults, but the expression of genes related to IFN signal, NLRP3 inflammasome, and other congenital pathways was higher in children, suggesting that the innate immune response in children is stronger in the early stage, which may be beneficial to better clinical results in children. However, the specific role of NLRP3 inflammasome in children with COVID-19 is still unknown (193). Notably, COVID-19 is not a common cause of ALI in children because they have fewer ACE receptors (194). The causes of age-related differences in early immune response in SARS-CoV-2 are still being studied, and there is no definite evidence that the activation of NLRP3 inflammasome is beneficial to COVID-19 in children. Considering the limited direct data on NLRP3 inflammasome and SARS-CoV-2 infection, the recent incidence of this new pathogen and its global impact need to be further studied. In adult studies, severe COVID-19 is the focus of research. Studies have confirmed that NLRP3 is involved in the severity of SARS-CoV. Anti-NLRP3 therapy shows obvious efficacy in clinical trials and animal models of SARS-CoV and SARS-CoV-2, strongly indicating that NLRP3 is the pivotal regulator of severe COVID-19 (153). As previously mentioned, gram-negative and gram-positive bacteria activate NLRP3/caspase-1 signaling, ultimately leading to cell rupture releasing IL-1β and IL-18. In summary, the bacteria and viruses that cause CAP can also induce pyroptosis through the NLRP3 inflammasome pathway, which is crucial to the damage of airway cells and the formation of acute lung inflammation (148). Inhibition of pyroptosis may be one of the future treatments for CAP.

The timing of CAP treatment is critical, as complications such as ARDS and respiratory failure may lead to poor prognosis in individuals (153). As COVID-19 is still prevalent all over the world, there is an urgent need to develop treatments to improve outcomes for patients with severe COVID-19. Therefore, in the context of an urgent need for drug discovery, it is valuable to use the known NLRP3 inflammasome as an intermediary of inflammatory signals for future pathogenesis exploration and treatment development.

## Conclusion

Pyroptosis is a way of programmed cell death, which normally participates in the immune process of the body, but excessive pyroptosis will cause a large number of cell death and have harmful effects on the body. Among the traditional signaling pathways, NLRP3 and caspase-1 play the most extensive role in respiratory diseases. They play an important role in the pathogenesis of respiratory diseases such as ALI, bronchial asthma, and BO by participating in acute and chronic respiratory inflammation, cell injury, and airway repair. This review summarizes the latest progress and experimental studies of pyroptosis in respiratory diseases. Studies have shown that for many respiratory diseases described in this review, some medicinal plants and inhibitors, by inhibiting the expression of NLRP3 inflammasome or caspase-1, can inhibit the pathway of pyroptosis, reduce the degree of pyroptosis, reduce inflammatory reaction and cell damage, to slow down the progression of the disease and improve the prognosis, this method may become a new target for the treatment of respiratory diseases. However, the mechanisms of pyroptosis have not been fully elucidated, more studies are still in the vitro research stage, and more prospective clinical trials are needed to transform it into clinical practice, especially in pediatric patients. There is still a long way to go, and more studies are needed to explore and understand the molecular mechanism and treatment prospects of pyroptosis, as well as possible new treatment ideas.

## Author contributions

Conceptualization: YL. Writing – original draft: JS. Writing - Review & Editing: YL. Data Curation: JS and YL. All authors contributed to the article and approved the submitted version.

## Conflict of interest

The authors declare that the research was conducted in the absence of any commercial or financial relationships that could be construed as a potential conflict of interest.

## Publisher’s note

All claims expressed in this article are solely those of the authors and do not necessarily represent those of their affiliated organizations, or those of the publisher, the editors and the reviewers. Any product that may be evaluated in this article, or claim that may be made by its manufacturer, is not guaranteed or endorsed by the publisher.
